# Evaluation of dietary supplementation of *Ascophyllum nodosum* and *Lithothamnium calcareum* as functional algae in F4+ *Escherichia coli* challenged piglets

**DOI:** 10.3389/fvets.2024.1430347

**Published:** 2024-09-06

**Authors:** Matteo Dell’Anno, Sara Frazzini, Serena Reggi, Irene Ferri, Elena Scaglia, Linda Schiasselloni, Alessia Inglesi, Federica Riva, Nicole Verdile, Rolando Pasquariello, Luciana Rossi

**Affiliations:** ^1^Department of Veterinary Medicine and Animal Sciences—DIVAS, University of Milan, Lodi, Italy; ^2^Department Civil, Environmental, Architectural Engineering and Mathematics—DICATAM, University of Brescia, Brescia, Italy; ^3^Department of Agricultural and Environmental Sciences—Production, Landscape, Agroenergy–DISAA, University of Milan, Milan, Italy

**Keywords:** seaweeds, brown algae, red algae, gut health, antioxidants, alternatives to antimicrobials, functional ingredients, enterotoxigenic *Escherichia coli*

## Abstract

**Introduction:**

Despite progress in reducing antimicrobial use in the veterinary field, it is crucial to find alternatives to preserve effectiveness and limit antimicrobial resistance. In pig farming, pathogenic strains of *E. coli* are the main cause of gastrointestinal disorders and antibiotic use. In this field, algae represent an innovation in animal nutrition that aligns with livestock sustainability principles and provide a high content of functional molecules.

**Aim:**

The aim of this study was to evaluate the impact of an innovative dietary combination of *Ascophyllum nodosum* and *Lithothamnium calcareum*, on growth, duodenum gene expression, jejunum intestinal morphology, and serum oxidative status in F4+ *Escherichia coli* challenged piglets.

**Materials and methods:**

Forty-eight weaned pigs, aged 28 ± 2 days, were divided into two groups (*n* = 24 pigs/group): the control group was fed a commercial diet (CTRL), while the seaweeds group was fed a commercial diet supplemented with 1.5% *A. nodosum* and 0.5% *L. calcareum* for 27 days (ALGAE). After 13 days, 50% of animals in each group were challenged with a single dose of 10^8^ CFU/dose of *E. coli* F4+, resulting in two infected groups (CTRL+ and ALGAE+, *n* = 12 pigs/group). Growth performance was assessed by measuring the individual body weight. At day 27, from six animals/group duodenum and jejunum sections were sampled for gene expression analysis via qRT-PCR and histological evaluation.

**Results and discussion:**

The results indicated a significantly higher body weight in the ALGAE+ group compared to CTRL+ after 7 days post-challenge (*p* < 0.0001). Jejunum morphology revealed lower villus height, villus width and villus height/crypt depth ratio in CTRL+ compared to ALGAE+ (*p* < 0.05) suggesting a protective effect of seaweeds on gut health.

**Conclusion:**

In conclusion, algae mixture exerted a protective effect against intestinal damage from *E. coli* F4+ infection proposing *A. nodosum* and *L. calcareum* supplementation as interesting strategy to support animal growth, enhance health and reduce antibiotic treatments in weaned piglets.

## 1 Introduction

In swine farming, weaning is one of the most critical phases in the productive life of a pig. During this period, piglets, with immature immune and digestive system, undergo several stressors such as mother’s separation, new environment and groups, and the gradual loss of passive immunity (1). This condition can lead to a significant activation of the hypothalamic–pituitary–adrenal axis, resulting in the release of glucocorticoids that increase the susceptibility to pathogenic invasion (2). Thus, gastrointestinal disorders are frequently registered during this period leading farmers and veterinarians resort to antibiotic treatments to limit their detrimental effect on animal health (3). Post-weaning diarrhea (PWD) is mainly caused by pathogenic *Escherichia coli* serotypes, causing high morbidity and mortality rates posing substantial concerns for farm profitability (4). Among pathogenic *E. coli*, enterotoxigenic *E. coli* (ETEC) are a group of bacteria characterized by important virulence factors involved in enterocyte adhesion, such as F4 fimbria, and toxins production causing enterotoxaemia (5). These pathogens are one the most prevalent causes of severe and watery diarrhea in piglets (6).

The overuse and misuse of antibiotics during the last 30 years has fostered the development of antibiotic resistance in several gut-associated *E. coli*, thereby posing therapeutic challenges and raising global concerns regarding antibiotic resistance (7). Consequently, there is an imperative demand for alternatives to antimicrobials that not only support animal health but also align with European policies encouraging the reduction of antibiotic use in food-producing animals (8). Although the relationship between the reduction in antibiotic use in food-producing animals and the circulation of resistant bacteria in humans is not yet well-defined (9), the scientific community generally agrees on the need to address the issue through a One Health approach following the principles of “reduce, replace, and re-think” (10–12).

The identification of holistic strategies, alternatives to antibiotics, aimed at improving intestinal health, has become a focus not only for farmers but also for the entire livestock system (13). Specifically, intensive farms have proposed and adopted multi-level solutions that collectively contribute to improve animal health and reduce reliance on antimicrobials. There has been increased attention to hygiene and health aspects, including targeted sanitation interventions, reduced animal density, more specific and timely therapies, as well as targeted vaccination prophylaxis and the use of functional nutritional strategies. Feed, in close contact with the gastrointestinal tract, is the primary entry point for pathogens into the host, and constantly in a state of immune stimulation, which significantly influence the animal’s health (14). Moreover, there has been a shift in the perception of nutrition that should not only meet nutrient requirements but also promote animal welfare, prevent diseases, and respect the environment (15). In this regard, functional ingredients are gaining raising attention in the zootechnical sector, not only for their nutritional effects but also because they are relevant to animal health, reducing the risk of disease onset (16). In this scenario, algae represent as a natural source of novel compounds with interesting bioactivities as functional ingredients in pig’s diets (17, 18).

Marine algae are gradually gaining interest in the European agri-food system for both human and animal nutrition, with positive reflects on health, and environmental protection. Macroalgae, also known as seaweeds, cultivation is a low-cost technology that does not require agricultural soil (19). Considering the agro-zootechnical scenario in the coming years, feed formulations including novel resources with innovative raw materials or new additives will be a decisive step toward sustainable animal production (20–22). *A. nodosum* is a brown macroalga typically from Europe and North East Atlantic Ocean, particularly common along sheltered, intertidal, rocky shores throughout Norway, Iceland, Arctic Canada, Greenland, and Portugal (23). *A. nodosum* has been one of the most studied seaweeds in recent years for animal nutrition. It is rich in minerals such as potassium and iodine (24) and contains polyphenols and phlorotannins, counted among bioactive compounds (17). *Phymatolithon calcareum*, commonly known as *L. calcareum* is a calcareous marine alga belonging to Rhodophyta typically abundant in the Mediterranean Sea, the Gulf of California, the Atlantic coast from northeastern Canada to the eastern Caribbean, and from Norway to the Canary Islands (25). *L. calcareum* is mainly composed of calcium carbonate, with recognized buffer ability for stabilizing rumen pH, milk yield, and feed efficiency compared to sodium bicarbonate (26). In addition, the sulfated polysaccharides form *Lithothamnium* could reduce the inflammation in humans and mice (27). Recent studies have shown promising results regarding the use of seaweeds in pig feeding, ranging from performance improvement to enhanced health status and the inhibition of pathogenic microorganisms (28). Building upon promising results observed in a previous study where the several algae have been evaluated for their functional characteristics *in vitro*, the combination of *A. nodosum* and *L. calcareum* revealed possible synergistic effect for antioxidant activity (29). In addition, the combination of these algae species showed a prebiotic effect *in vitro* when added to the medium of *Lactiplantibacillus plantarum* and *Limosilactobacillus reuteri* cultures as probiotic species isolated from swine (30).

In light of these findings, we hypothesize that the dietary supplementation of the combination of *A. nodosum* and *L. calcareum* could positively impact animal health preventing the infection by pathogenic *E. coli* improving animal performance and gut health. Thus, this paper targets to unravel the combined effect of this innovative blend as functional ingredients in weaned piglets. Specifically, the aim of this study was to evaluate the impact of an innovative dietary combination of *A. nodosum* and *L. calcareum*, on growth performance, challenger strain shedding, protein digestibility, duodenum gene expression, jejunum morphology, and serum oxidative status in F4+ *E. coli* challenged piglets.

## 2 Materials and methods

### 2.1 Algae characterization

*A. nodosum* and *L. calcareum* samples were analyzed in duplicate for the content of principal nutrients according to the Official Methods of Analysis (22). In particular, freeze-dried algae meals were analyzed for their content of dry matter (DM) drying samples in a forced air oven at 65°C until reaching stable weight (AOAC method 930.15). Crude protein (CP) using the Kjeldahl method (with a nitrogen conversion factor of 6.25; AOAC method 2001.11). Ether extract (EE) was quantified using a Soxtec extractor (SER 148, VELP Scientifica Srl, Italy) by samples submersion and boiling in petroleum ether (Randall method; AOAC 2003.05). Crude fiber (CF) was obtained according to the Weende method using the filtering bags technique (AOCS Ba 6a-05). Ashes were obtained by incinerating algae meals at 550°C in a muffle furnace (AOAC method 942.05).

### 2.2 Animals, experimental design, and treatments

All experimental procedures have been approved from the Animal Welfare Organization of University of Milan and the Italian Ministry of Health (authorization n° 884/2021-PR). Forty-eight crossbred (Landrace × Large White) weaned piglets (28 ± 2 day-old; 6.89 ± 0.81 kg) were housed at the Experimental Zootechnical Center of the University of Milan. Animals were identified by ear tags and housed in individual pens under controlled conditions (29–27°C; 50–60% of relative humidity). Piglets had free access to water, feed, and environmental chewable enrichments according to Italian and European regulations (31) (D.lgs 122/2011).

Experimental diets were formulated with Plurimix Software to satisfy the nutritional requirements of weaned piglets, provided by Ferraroni S.p.A. (Bonemerse, Italy) (Table 1). At housing, animals were fed commercial diets for an adaptation period of 7 days. After the adaptation period, animals were divided into two experimental groups balanced per weight and sex: control group (CTRL, 24 piglets) fed with the same commercial diet for the entire trial, and treatment group (ALGAE, 24 piglets) fed with the same commercial diet supplemented with 2% of algae mixture (1.5% of *A. nodosum* and 0.5% of *L. calcareum*) for 27 days of trial. The treatment diet was manufactured by mixing 2 kg of seaweeds blend with 100 kg of feed in a horizontal mixer for 20 min in order to ensure the correct dispersion of agal meals. Seaweeds were purchased by Italfeed Srl (Milan, Italy) as feed ingredients for experimental diet’s preparation. Sub-samples from CTRL and ALGAE produced batches were collected for the analysis of DM, CP, EE, CF, and ashes in duplicate according to the aforementioned methodology.

**Table 1:** Experimental diet formula of control (CTRL) and treatment supplemented with *A. nodosum* and *L. calcareum* (ALGAE).

Ingredients, % (as Fed basis)	CTRL	ALGAE
Barley, meal	24.84	24.34
Wheat, meal	23.74	23.27
Soybean, meal	7.00	6.86
Corn, flakes	6.61	6.48
Corn, meal	6.55	6.42
Wheat, flakes	6.31	6.18
Soy protein concentrate	5.43	5.32
Dextrose monohydrate	2.99	2.93
Sweet milk whey	2.97	2.91
Soybean protein concentrate	2.97	2.91
Soy oil	1.98	1.94
Beet pulp	1.93	1.89
Coconut oil	1.07	1.05
Plasma, meal	1.00	0.98
Acidifiers mix^1^	0.79	0.77
L-Lysine	0.60	0.59
Herring, meal	0.59	0.58
Dicalcium phosphate	0.51	0.50
Vitamins and minerals premix^2^	0.50	0.49
Benzoic acid	0.50	0.49
DL-Methionine	0.22	0.22
L-Threonine	0.22	0.22
Tri-and Di-butyrate	0.20	0.20
Sodium chloride	0.15	0.15
L-Valine (96.5%)	0.11	0.11
Calcium carbonate	0.08	0.08
Flavor	0.05	0.05
Enzymatic mix^3^	0.05	0.05
L-Tryptophan	0.04	0.04
*A. nodosum*	-	1.50
*L. calcareum*	-	0.50
Calculated chemical composition		
Crude protein (%)	17.50	17.26
Crude lipid (%)	4.80	4.73
Crude fiber (%)	2.90	2.99
Ashes (%)	4.30	5.06

^1^Benzoic acid, citric acid, and folic acid. ^2^Additives per Kg: Vitamins, pro-vitamins and substances with similar effect. Vitamin A (Retinyl acetate) 15000.00 UI, Vitamin D3 (cholecalciferol) 2000.00 UI, Vitamin B1 (Thiamine mononitrate) 2.00 mg, Vitamin B12 (Cyanocobalamin) 0.03 mg, Vitamin B2 (Riboflavin) 5.00 mg, Vitamin B6 (pyridoxine hydrochloride) 3.50 mg, Vitamin C (sodium calcium ascorbyl phosphate) 150.00 mg, Vitamin E (all-rac-alpha-tocopheryl acetate) 120.00 mg, Vitamin K3 (menadione nicotinamide bisulphite) 2.00 mg, Betaine hydrochloride 1000.00 mg, Biotin 0.20 mg, Choline chloride 300.00 mg, Niacinamide 30.00 mg, Butyl hydroxytoluene (BHT) 0.30 mg, Propyl gallate 0.15 mg, Sepiolite 576.50 mg, Bentonite 4900.00 mg, Calcium D-pantothenate 15.00 mg, Copper (Copper Oxide-I) 100.00 mg, Iodine (Calcium iodate anhydrous) 1.00 mg, Iron (Iron-II sulfate monohydrate) 120.00 mg, Manganese (Manganese-II oxide) 50.00 mg, Selenium (Sodium selenite) 0.35 mg, and Zinc (Zinc oxide) 100.00 mg. ^3^6-phytate, Endo1,3(4)-beta-glucanated, Endo-1,4-beta-xylanation.

### 2.3 Experimental challenge

Thirteen days after feeding experimental diets (day 13), 24 piglets (12 animals/group) were orally challenged with enterotoxigenic F4+ *Escherichia coli* streptomycin resistant, from the strain collection of the Department of Veterinary Medicine and Animal Sciences of University of Milan. The challenge procedures were performed according to the translational model developed by Rossi et al. (32). Briefly, piglets were injected with azaperone (2 mL/pig, Stresnil™, Janssen Cilag SpA, Milan, Italy) and 30 mL of 10% bicarbonate solution was orally administered to increase the challenger strain survival rate in the gastric environment. After 10–15 min, a single dose of 5 mL of 1 × 10^8^ CFU of an overnight culture in Luria Bertani (LB) medium of F4+ *E. coli* was orally delivered for the infected groups. Uninfected groups were orally delivered with sterile LB culture media. Infected piglets were physically separated from uninfected animals and biosafety protocols have been adopted to avoid the pathogenic transfer among groups. The experimental challenge generated two sub-groups: infected control (CTRL+) and infected algae (ALGAE+) group. Uninfected piglets were identified with CTRL− and ALGAE−, according to the provided diet.

### 2.4 Zootechnical performance and sample collection

**Figure 1:**
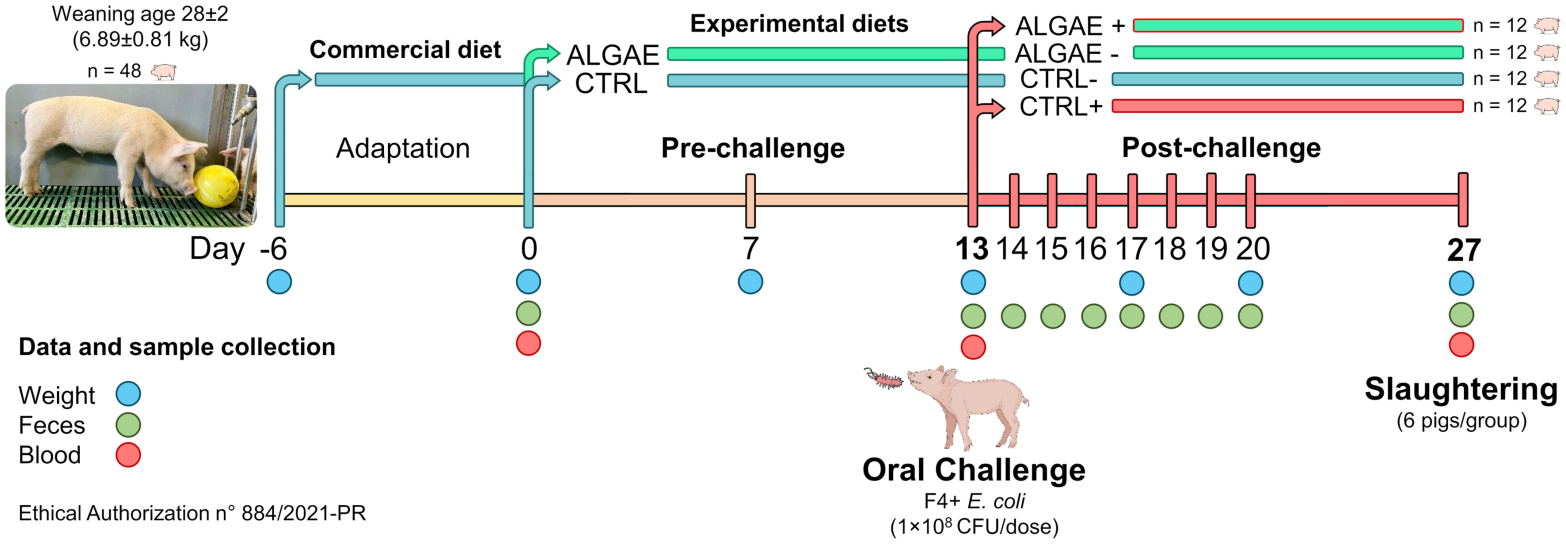
Experimental design and sample collection.

Figure 1 resumes the experimental design and sample collection. In detail, piglets were individually weighted at day 0, 7, 13, 20, and 27 and feed refuse was measured three times per week to evaluate the average body weight gain, average feed intake, and calculate the feed conversion ratio. Fecal samples were collected at 0, 13, 14, 15, 16, 17, 18, 19, 20, and 27 days for the evaluation of challenger strain shedding and fecal plate counting by microbiological and molecular analysis. Blood samples were collected at 0, 13, and 27 days of trial with vacuum tubes without anticoagulants. After 27 days from the administration of experimental diets 50% of animals (six pig/group) were slaughtered and intestinal section of duodenum and jejunum were collected for histological and gene expression analysis. Daily, health conditions were checked, and fecal consistency was evaluated using the fecal score according to a four-levels scale following Rossi et al. (32), considering moderate diarrhea for a score = 2 and severe diarrhea = 3. Fecal color was evaluated with a three-level scale: 0 = yellowish color; 1 = greenish color; 2 = brown color considering pathological a score < 2.

### 2.5 *Escherichia coli* F4+ shedding

The shedding of the challenger strain was assessed using the plate counting technique. Specifically, 1 g of fresh feces was diluted 1:10 (w/v) in physiological solution (0.9% NaCl, Sigma Adrich, St. Louis, MO, United States) and plated in triplicate on Petri dishes containing a selective medium, Violet Red Bile Glucose Agar (VRBG agar, Liofilchem Srl, Roseto degli Abruzzi, Teramo, Italy), supplemented with streptomycin (50 μg/mL) for each dilution. After incubation for 24 h at 37°C, coliforms appeared as red colonies. The number of colony-forming units per gram of feces was then measured.

The abundance of F4+ *E. coli* within the fecal samples from day 14 to day 20 was also estimated using qPCR. Genomic DNA of *E. coli* was extracted (33) and used to create a standard curve by linear correlation between the log of the number of copies and CT values. The qPCR curve was obtained by amplifying serial dilutions of genomic DNA (1:10) in triplicate (*R*^2^ = 0.99). The qPCR was performed using the CFXOPUS 96 (Bio-Rad Laboratories, Inc., Hercules, CA, United States) and SsoAdvanced Universal Syber Green Supermix (Bio-Rad Laboratories, Inc., Hercules, CA, United States) with primers designed for the amplification of FaeG gene (Acc. N°: AY437806; FW: GGTTCTGAACTCTCGGCTG and RV: AGAACCTGCGACGTCAACAA; amplicon size 135 base pairs). The specificity of the primers at the species level was confirmed by performing BLAST searches of the Gene Bank Database (NCBI BLAST). The thermal cycling condition consisted of an initial step at 95°C for 2 min, followed by 40 cycles at 95°C for 10 s and 60°C for 30 s. The specificity of the primers was confirmed by melting curve analysis under the following thermal conditions: 95°C for 10 s, with an increment of 0.5°C per 5 s from 65 to 95°C. Genomic DNA was extracted from feces using the QIAamp PowerFecal Pro DNA Kit (QIAGEN, Hilden, Germany), following the manufacturer’s instructions. For the qPCR, we used 50 ng of genomic DNA and amplified it under the same conditions as the calibration curve.

### 2.6 Microbiological analysis of fecal samples

Fecal samples from 0, 13, and 27 days were analyzed for the enumeration of total, lactic acid and coliform bacteria through specific culture agar media (Plate Count Agar—PCA, De Man Rogosa and Sharpe Agar—MRS, Violet Red Bile Broth Agar—VRBA, respectively; Liofilchem Srl, Roseto degli Abruzzi, Teramo, Italy) (34). 1 g of fecal samples was diluted and homogenized 1:10 (w/v) in sterile 0.9% NaCl solution and centrifuged (3,000 rpm, 10 min at room temperature). 1 mL of the supernatant was serially diluted tenfold and plated using the inclusion method for PCA, MRS, and VRBA media. After 24 h of incubation at 37°C, visible colonies were counted for VRBA and 48 h for PCA and MRS. The lactic acid bacteria:coliform ratio was calculated. The results were expressed as log_10_ of colony forming units per gram of fresh feces (log_10_ CFU/g).

### 2.7 Antioxidant barrier and oxidative status of serum samples

Blood samples were centrifuged at 3,000 rpm for 10 min at room temperature to obtain serum (35). Each sample was then assessed for antioxidant barrier status through the Oxy-Adsorbent test (Diacron Srl, Grosseto, Italy) and oxidative status using the dROMs test (Diacron Srl, Grosseto, Italy), following the manufacturer’s instructions. Endpoint absorbances were measured at 546 and 505, respectively, using a UV–Vis spectrometer (V630, UV–Vis, Jasco GmBH, Pfungstadt, Germany).

### 2.8 RNA extraction from duodenum samples

Total RNA was isolated from duodenum samples using the TRIzol (Sigma-Aldrich, St. Louis, MO, United States) method following the manufacturer’s instructions (36). Briefly, the tissues were homogenized in 1 mL of TRIzol using a rotor-stator system (Ultra Turrax, IKA WERKE, Staufen, Germany). The RNA concentration and purity (1.6–2.06) was established by measuring absorbance at 260 and 280 nm wavelength with a spectrophotometer (BioPhotometer, Eppendorf, Hamburg, Germany). The samples were stored at −20°C until use. According to the manufacturer’s instructions, 1 μg of total RNA from all the samples was reverse transcribed using iScript™ cDNA Synthesis kit (Bio-Rad Laboratories, Hercules, CA, United States). The reverse transcription was performed in a 20 μL reaction mixture according to the following program: step 1, incubation at 25°C for 5 min; step 2 at 46°C for 20 min; and step 3 at 95°C for 1 min.

### 2.9 Primers design

Pig messenger sequences and genomic sequences were obtained by GenBank, the NIH genetic sequence database and the accession numbers of all the sequences are listed in Table 2 (35). Messenger sequences were aligned with genomic DNA sequences using the BLAST software of Gene Bank Database to identify exon/intron boundaries (NCBI BLAST). Our primer pairs were designed using Primer3, a free software, with the support of the Primer-BLAST on the mRNA sequences of target genes and selected to produce amplicons spanning two exons. The suggested primers were checked to achieve the following criteria: amplicon length (50–180 bp), melting temperature (Tm), primer length (between 18 and 24 bp), %GC content, self-complementary, primer-dimer and hairpin formation, degree of degeneracy, 5′ end stability, and 3′ end specificity. Primers were purchased from Eurofins (Eurofins Genomics, Germany). Then, each primer pair was tested with qualitative PCR on cDNA extracted from intestinal tissue. PCR products were separated by electrophoresis on a 2% agarose gel. Only PCR products of the expected size were cut, purified by QIAquick Gel Extraction Kit using a Microcentrifuge (QIAGEN, Hilden, Germany), and sequenced for appropriate amplicon size and high-quality sequence.

**Table 2:** Oligonucleotides sequences used for the qRT-PCR assay of duodenum samples.

Gene^1^	Nucleotide sequence	Accession number
COX1	Fw: GGAGCGGGTACTGGATGAAC	EF568726
	Rv: CACCTGCAAGGGTGTAGGGAG	
COX2	Fw: AAGACGCCACTTCACCCATC	AF304201
	Rv: TCCATTGTGCTAGTGTGTGTCA	
IL-10	Fw: TGAAGAGTGCCTTTAGCAAGCTC	NM_214041.1
	Rv: CTCATCTTCATCGTCATGTAGGC	
IL-8	Fw: TGCCTTCTTGGCAGTTTTCCT	NM_213867
	Rv: TGGGGTCCACTCTCAATCACT	
OCLN	Fw: GTCCACCTCCTTATAGGCCTGATG	NM_001163647
	Rv: CGCTGGCTGAGAAAGCATTGG	
SOD1	Fw: GTGCAGGGCACCATCTACTTC	NM_001190422
	Rv: GATCACCTTCAGCCAGTCCTT	
TGF-β	Fw: TGATGTCACCGGAGTTGTGC	NM_214015.2
	Rv: GGCCAGAATTGAACCCGTTA	
NQ01	Fw: ATCACAGGTAAACTGAAGGACCC	NM_001159613
	Rv: GCGGCTTCCACCTTCTTTTG	
ACT-β	Fw: CTACGTCGCCCTGGACTTC	DQ845171
	Rv: GCAGCTCGTAGCTCTTCTCC	

^1^COX1, Cytochrome c oxidase subunit I; COX2, Cytochrome c oxidase subunit II; IL-10, Interleukin 10; IL-8, Interleukin-8; OCLN, Occludin; SOD1, Superoxide dismutase 1; TGF-β, Transforming growth factor-beta; NQ01, NAD(P)H quinone dehydrogenase 1; ACT-β, Actin beta.

### 2.10 Real-time qRT-PCR

The cDNA obtained from each sample was used as a template for Real-Time PCR in an optimized 20 μL reaction volume in Hard-Shell® 96-Well PCR Plates (Bio-Rad Laboratories, Hercules, CA, United States) using SsoAdvanced universal SYBR® Green Supermix (Bio-Rad Laboratories, Hercules, CA). Each plate contained duplicates of each cDNA sample, 2X SYBR Green supermix and primers at 250 nM each. A duplicate no-template control (NTC) was included in each experiment as well as a calibrator sample. RT-qPCR was carried out in the CFX96 Opus Real-Time Detection system (Bio-Rad, Hercules, CA, United States). The standard two-step qPCR cycling conditions were as follows: pre-denaturation of 95°C for 30 s, 40 cycles of denaturation of 95°C for 15 s, and an annealing/extension step at 60°C for 30 s. When the cycle threshold (Ct) values of target genes were > 37, the results were deemed negative. Melting curve analysis to confirm target-specific amplification was performed after PCR reactions according to the protocol. A control negative sample, selected as a healthy piglet from CTRL-group, was used as the calibrator sample. Quantification of the expression gene was determined with the calculation of 2^−ΔΔCT^. ACT-β has been included as housekeeping genes according to Caprarulo et al. (35).

### 2.11 Morphological analysis of duodenum and jejunum sections

After the slaughtering, 2 cm of duodenum and jejunum were collected and immediately submerged in 10% neutral buffer formalin (37). Following 24 h of fixation at room temperature, the intestinal segments were dehydrated in graded alcohols, cleared with xylene and embedded in paraffin. The paraffinic blocks were cut into 5 μm sections using a rotating microtome (MR2258, Histo-line laboratories). They were dewaxed and re-hydrated, and they were stained with hematoxylin/eosin to evaluate the morphological features using a ZEISS Axio Zoom.V16 microscope (Carl Zeiss AG, Oberkochen, Baden-Württemberg, Germany) for imaging. The morphometry was quantified by measurements of the villi and crypts. A minimum of 20 well-oriented and intact villi and crypts were selected per image. The villus length was measured from the tip of the villus to the villus–crypt junction. The crypt depth was defined as the depth of the invagination between adjacent villi. The measurements were made using the ZEISS ZEN Microscopy Software.

### 2.12 Statistical analysis

Data were analyzed using GraphPad Prism ver. 9.0. Normality of the distribution of data was checked using the Kolmogorov–Smirnov test assuming normal distribution for a *p* value >0.05. Zootechnical performance, challenger strain qPCR, fecal bacterial counts, and serum oxidative status results were analyzed using a mixed model which included the fixed effect of treatment, time, their interaction (treatment × time), and subject was included as random effect. *Post-hoc* comparisons were performed using the Sidak correction, which is well-suited for mixed models and provides a balanced approach to controlling the family-wise error rate while maintaining statistical power, since Tukey was not appropriate for the assumptions of this model. Diarrhea frequency was calculated by dividing the number of clinical manifestations by total observations performed for the considered period for each group. Statistical differences in diarrhea frequency were evaluated by transforming the fecal score values into a dichotomous variable (presence/absence; presence for a score > 1) for testing the differences between observed and expected frequencies through the chi-squared (χ^2^) test. Histological measurements, fecal parameters, and protein digestibility results were analyzed through one-way ANOVA, and pairwise comparisons were evaluated with Tukey’s test. Data from qRT-PCR were analyzed using a Kruskal-Wallis test for comparing median of different groups due to the limited number of samples per group. The means or medians were considered statistically different for *p* ≤ 0.05. The results have been presented as means ± standard error or median and range (minimum – maximum) depending on the data distribution.

## 3 Results

### 3.1 Nutritional composition of algae and experimental diets

*A. nodosum* and *L. calcareum* meals revealed high ash contents (25.33 and 92.75% as fed basis, respectively), followed by the content of crude fiber contents (8.92 and 2.91% as fed basis, respectively), consistent with previous literature studies. The experimental diets showed a balanced composition, and seaweeds mixture supplementation did not affect the feed formula which aligned with the nutritional requirements of post-weaning pigs (Table 3) (38).

**Table 3:** Chemical composition of seaweeds and experimental diets prepared for the experimental trial.

Analyte (%, as fed basis)	*A. nodosum*	*L. calcareum*	CTRL	ALGAE
Moisture	8.56	0.40	6.81	6.62
CP	6.93	0.21	18.38	18.35
EE	1.79	0.27	4.51	4.63
*CF*	8.92	2.91	2.74	2.66
Ash	25.33	92.75	4.82	4.89

CP, Crude protein; EE, Ether extract; CF, Crude fiber; CTRL, Control diet; ALGAE, Commercial diet supplemented with 1.5% of *A. nodosum* and 0.5% of *L. calcareum*.

### 3.2 Zootechnical performance

All piglets showed comparable performance during the pre-challenge period with no difference in CTRL and ALGAE groups (Table 4). Even if the effect of time × treatment was significant for BW during the pre-challenge, *post-hoc* comparisons did not reveal any difference between experimental groups in this period. From day 13 to day 27, the four groups revealed significant differences in terms of performance (*p* < 0.0001). BW of CTRL+ was significantly lower at 20 days compared to ALGAE+ (*p* < 0.05). During the period from 13 to 20 days the average daily gain was significantly decreased in ALGAE+ and CTRL+ compared to other groups (*p* < 0.05). In particular, ALGAE+ revealed a lower ADG compared to CTRL+ from 13 to 17 days. From 17 to 20 days of trial CTRL+ registered a weight loss resulting in negative ADG compared to other groups, meanwhile ALGAE+ showed a significant higher growth if compared to all experimental groups. The feed intake registered a high individual variability without showing significant difference during both pre-and post-challenge periods. FCR resulted significantly higher in CTRL+ from the period from 13 to 20 days compared to CTRL−, ALGAE−, and ALGAE+ groups (*p* < 0.05).

**Table 4:** Zootechnical performance during the pre-challenge (0–12 days) and post-challenge (13–27 days) periods for control (CTRL) and algae mixture (ALGAE) supplemented groups divided per unchallenged (−) and challenged (+) groups during the post-challenge period after day 12.

	Period	Groups				*p* values		
BW (kg)	Pre-challenge	CTRL	ALGAE			Time	Trt	Time × Trt
	d 0	7.66 ± 0.18	7.68 ± 0.19			<0.0001	0.2912	0.0332
	d 7	8.18 ± 0.29	8.80 ± 0.26					
	Post-challenge	CTRL−	ALGAE−	CTRL+	ALGAE+			
	d 13	9.72 ± 0.79	10.38 ± 0.48	10.17 ± 0.37	10.57 ± 0.39	<0.0001	0.4839	<0.0001
	d 17	13.89 ± 0.53	14.08 ± 0.65	13.14 ± 0.41	12.30 ± 0.51			
	d 20	14.58 ± 0.61^ab^	15.08 ± 0.70^ab^	13.04 ± 0.53^b^	15.66 ± 0.51^a^			
	d 27	19.66 ± 0.64	19.74 ± 0.76	18.28 ± 0.68	18.89 ± 0.62			
ADG (g/day)	Pre-challenge	CTRL	ALGAE					
	d 0–7	73.38 ± 31.08	78.57 ± 50.00			0.0478	0.7029	0.6567
	d 7–13	276.19 ± 37.33	208.33 ± 75.00					
	Post-challenge	CTRL−	ALGAE−	CTRL+	ALGAE+			
	d 13–17	1043.75 ± 47.59^a^	925.00 ± 74.87^a^	740.91 ± 60.80^b^	433.33 ± 56.10^c^	<0.0001	0.0003	<0.0001
	d 17–20	227.78 ± 70.69^a^	330.56 ± 128.28^a^	−33.33 ± 121.02^b^	1119.45 ± 90.22^c^			
	d 21–27	726 ± 30.09	666.67 ± 74.34	749.35 ± 52.70	461.91 ± 42.14			
ADFI (g/day)	Pre-challenge	CTRL	ALGAE					
	d 0–12	535 ± 35	547 ± 33				0.7726	
	Post-challenge	CTRL−	ALGAE−	CTRL+	ALGAE+			
	d 13–20	640 ± 81	815 ± 82	699 ± 51	688 ± 52	0.4371	0.3027	0.3323
	d 21–27	789 ± 80	783 ± 56	624 ± 87	794 ± 71			
FCR (ratio)	Pre-challenge	CTRL	ALGAE					
	d 0–13	2.55 ± 0.52	2.53 ± 0.17				0.9738	
	Post-challenge	CTRL−	ALGAE−	CTRL+	ALGAE+			
	d 13–20	1.29 ± 0.11^a^	1.63 ± 0.16^a^	2.71 ± 0.59^b^	1.04 ± 0.08^a^	0.0278	0.1229	<0.0001
	d 21–27	1.11 ± 0.07	1.33 ± 0.16	0.9 ± 0.05	1.82 ± 0.13			

BW, Body weight; ADG, Average daily gain; ADFI, Average daily feed intake; FCR, Feed conversion ratio; Trt, Treatment effect; Time × Trt, Interaction effect between time and treatment.

During the adaptation period, the frequency of diarrhea registered was 2.08 and 2.78% of total observations in CTRL and ALGAE, respectively. From 0 to 7 days of trial, a significant increase in diarrhea frequency was observed in CTRL compared to ALGAE group (11.58% on total observations in CTRL and 5.12% of total observations in ALGAE; *p* < 0.05). During the period from 8 to 13 days only 4.35 and 3.33% of total feces observations showed signs of diarrhea in CTRL and ALGAE, respectively. Only one piglet was excluded from the trial due antibiotic treatment at day 6 of the trial after five consecutive days of diarrhea occurrence. From day 13 to 27 no diarrhea cases have been registered for the entire post-challenge period.

### 3.3 Challenger strain shedding and bacterial counts in faces

The fecal shedding plate counts in challenged groups showed 69.57% of positive feces to the challenger strain after 3 days from challenge (day 16) in ALGAE+ and CTRL+ groups, respectively. A fecal shedding positivity to *E. coli* > 20% lasted until day 18 of trial (5 days post-challenge).

**Figure 2:**
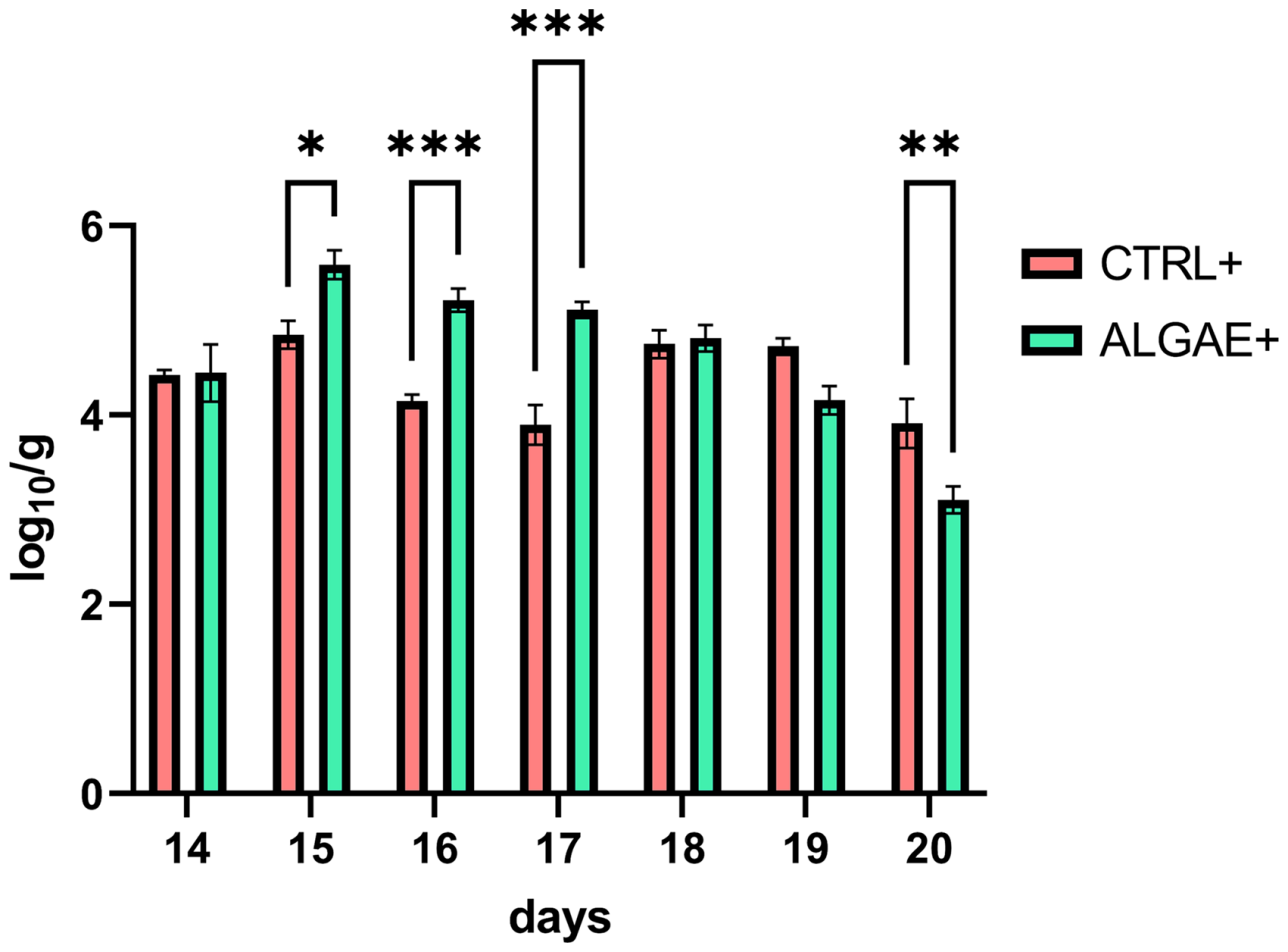
DNA abundance of challenger strain in feces during the 7 days after challenge (days 13–20) in infected control (CTRL+) and infected algae supplemented (ALGAE+) group. Data are presented as means ± standard error of log_10_/g of fresh feces. ^*, **, ***^Asterisks indicate that means are statistically different at *p* < 0.05, *p* < 0.01, and *p* < 0.001, respectively.

qPCR analysis revealed significant differences in the relative abundance of F4+ *E. coli* DNA in fecal samples (Figure 2) (*p* < 0.0001). In particular, ALGAE+ showed increased presence of bacterial DNA from day 15 to 17 compared to CTRL+ (*p* < 0.05). On the contrary, CTRL+ registered higher abundance of F4+ *E. coli* after 7 days post-challenge compared to ALGAE+ (day 20; *p* < 0.01).

Fecal bacterial count of total, lactic acid and coliform bacteria and their index did not register any significant difference for all experimental groups during the pre-and post-challenge periods (Table 5). Even if a significant treatment effect was observed for lactic acid bacteria count during the post-challenge (*p* = 0.0308), post-hoc comparisons did not reveal any differences among CTRL−, ALGAE−, CTRL+, and ALGAE+ groups in this period.

**Table 5:** Fecal bacterial count of total, lactic acid and coliform bacteria during the pre-and post-challenge period in control (CTRL) and algae mixture (ALGAE) supplemented groups divided per unchallenged (−) and challenged (+) groups during the post-challenge period after day 12.

	Period	Groups				*p* values		
Total bacteria (log_10_ CFU/g)	Pre-challenge	CTRL	ALGAE			Time	Trt	Time × Trt
	d 0	6.69 ± 0.28	6.73 ± 0.32				0.9222	
	Post-challenge	CTRL−	ALGAE−	CTRL+	ALGAE+	0.2151	0.0975	0.3789
	d 13	6.25 ± 0.18	6.67 ± 0.20	6.32 ± 0.32	7.01 ± 0.23			
	d 27	6.48 ± 0.29	6.17 ± 0.17	6.11 ± 0.22	6.73 ± 0.29			
Lactic acid bacteria (log_10_ CFU/g)	Pre-challenge	CTRL	ALGAE				0.3873	
	d 0	6.86 ± 0.22	6.59 ± 0.22					
	Post-challenge	CTRL−	ALGAE−	CTRL+	ALGAE+	0.0900	0.0308	0.6045
	d 13	6.63 ± 0.22	6.23 ± 0.20	6.56 ± 0.32	7.11 ± 0.23			
	d 27	6.16 ± 0.38	6.19 ± 0.27	6.06 ± 0.24	7.03 ± 0.23			
Coliform bacteria (log_10_ CFU/g)	Pre-challenge	CTRL	ALGAE				0.6582	
	d 0	3.56 ± 0.39	3.32 ± 0.34					
	Post-challenge	CTRL−	ALGAE−	CTRL+	ALGAE+	0.3195	0.2104	0.5681
	d 13	3.74 ± 0.36	3.05 ± 0.24	2.48 ± 0.34	3.01 ± 0.28			
	d 27	3.64 ± 0.55	3.24 ± 0.52	3.46 ± 0.41	3.07 ± 0.27			
Lactic acid/coliform bacteria ratio	Pre-challenge	CTRL	ALGAE				0.9874	
	d 0	2.29 ± 0.34	2.28 ± 0.40					
	Post-challenge	CTRL−	ALGAE−	CTRL+	ALGAE+	0.1539	0.6730	0.5360
	d 13	2.16 ± 0.42	2.15 ± 0.15	2.88 ± 0.29	2.56 ± 0.23			
	d 27	2.01 ± 0.34	2.15 ± 0.31	1.86 ± 0.32	2.31 ± 0.18			

CFU, Colony forming unit; Trt, Treatment effect; Trt × Time, Effect of the interaction between treatment and time. Data are presented means ± standard error.

### 3.4 Protein digestibility and fecal parameters

The fecal composition in terms of DM did not show any difference in experimental groups from 0 to 27 days of trial (Table 6). The fecal concentration of CP showed a significant increase in CTRL+ compared to ALGAE+ and CTRL− (*p* < 0.05). Consequently, the ATTD of protein component was significantly decreased in CTRL+ compared to CTRL− and ALGAE − groups at 27 days (*p* < 0.05).

**Table 6:** Fecal parameters measured at 0 and 27 days of trial in control (CTRL) and algae mixture (ALGAE) supplemented groups divided per unchallenged (−) and challenged (+) groups during the post-challenge period after day 12.

		Groups				*p* value
DM (%)	Pre-challenge	CTRL	ALGAE			
	d 0	26.39 ± 1.36	25.03 ± 2.06			0.5884
	Post-challenge	CTRL−	ALGAE−	CTRL+	ALGAE+	
	d 27	29.88 ± 1.17	28.77 ± 0.57	28.30 ± 0.58	28.41 ± 0.82	0.5211
CP (%)	Pre-challenge	CTRL	ALGAE			
	d 0	6.29 ± 0.47	7.92 ± 0.67			0.0577
	Post-challenge	CTRL−	ALGAE−	CTRL+	ALGAE+	
	d 27	5.70 ± 0.20^a^	5.92 ± 0.20^ab^	6.68 ± 0.33^b^	5.36 ± 0.19^a^	0.0054
ATTD of CP (%)	Pre-challenge	CTRL	ALGAE			
	d 0	96.07 ± 0.30	95.42 ± 0.30			0.1384
	Post-challenge	CTRL−	ALGAE−	CTRL+	ALGAE+	
	d 27	97.76 ± 0.15^a^	98.16 ± 0.06^a^	97.04 ± 0.12^b^	97.58 ± 0.10^ab^	<0.0001

Data are presented means ± standard error. ^a,b^Different lowercase letters indicate statistically significant differences among groups (*p* < 0.05).

### 3.5 Relative gene expression in duodenum

mRNA transcription in duodenum samples did not reveal any significant differences in the four considered groups at 27 days of trial (Table 7).

**Table 7:** Relative mRNA transcription of duodenum at 27 days of trial in control (CTRL) and algae mixture (ALGAE) supplemented divided per unchallenged (−) and challenged (+) groups.

	Groups				
Gene^1^	CTRL– (*n* = 5) median (min-max)	ALGAE– (*n* = 6) median (min-max)	CTRL+ (*n* = 6) median (min-max)	ALGAE+ (*n* = 6) median (min-max)	*p* value
COX1	0.83 (0.68–2.38)	1.23 (0.56–2.29)	1.00 (0.31–2.33)	0.64 (0.22–2.06)	0.6453
COX2	1.40 (0.65–4.20)	1.71 (0.59–4.33)	1.35 (0.45–2.78)	0.74 (0.23–5.25)	0.5229
IL-10	0.82 (0.11–5.03)	2.31 (0.57–2.88)	1.89 (0.84–8.09)	0.99 (0.25–4.48)	0.3653
IL-8	0.84 (0.10–2.97)	0.73 (0.02–2.72)	1.19 (0.31–3.53)	0.54 (0.10–5.20)	0.8741
OCLN	1.17 (0.13–2.18)	0.82 (0.02–1.35)	1.10 (0.64–1.85)	1.92 (0.24–2.06)	0.7616
SOD1	1.05 (0.07–2.53)	1.14 (0.03–1.64)	1.32 (0.48–2.55)	0.98 (0.31–5.07)	0.7300
TGF-β	0.69 (0.45–1.80)	1.54 (0.17–1.77)	1.57 (0.68–2.11)	0.71 (0.57–2.00)	0.2623
NQ01	1.42 (0.72–2.18)	1.18 (0.10–2.82)	1.21 (0.37–3.88)	2.66 (0.33–6.01)	0.4512

^1^COX1, Cytochrome c oxidase subunit I; COX2, Cytochrome c oxidase subunit II; IL-10, Interleukin 10; IL-8, Interleukin-8, OCLN, Occludin; SOD1, Superoxide dismutase 1; TGF-β, Transforming growth factor-beta; NQ01, NAD(P)H quinone dehydrogenase 1.

Data are presented as median value and range (minimum – maximum).

### 3.6 Serum oxidative status and antioxidant barrier

The serum samples of piglets at 0, 13, and 27 days did not register any significant differences in terms of UCARR and μM HClO/mL for the oxidative and antioxidant barrier status, respectively (Table 8). Even if a significant effect of the interaction between treatment and time was registered at day 13 for dROMs test, *post-hoc* comparisons did not display any difference among groups.

**Table 8:** Serum values of dROMs and Oxy Adsorbent test measured at 0, 13, and 27 days of trial in control (CTRL) and algae mixture (ALGAE) supplemented divided per unchallenged (−) and challenged (+) groups after day 12.

	Period	Groups				*p* value		
dROMs (UCARR)	Pre-challenge	CTRL	ALGAE			Time	Trt	Trt × Time
	d 0	191.08 ± 22.53	157.40 ± 15.08				0.2426	
	Post-challenge	CTRL−	ALGAE−	CTRL+	ALGAE+			
	d 13	279.82 ± 42.02	256.10 ± 44.99	211.18 ± 63.22	158.90 ± 32.01	<0.0001	0.2702	0.0145
	d 27	114.87 ± 14.01	114.94 ± 9.94	166.48 ± 11.46	138.74 ± 5.94			
Oxy Adsorbent (μM HClO/mL)	Pre-challenge	CTRL	ALGAE					
	d 0	318.29 ± 40.34	249.57 ± 32.77				0.2155	
	Post-challenge	CTRL−	ALGAE−	CTRL+	ALGAE+			
	d 13	204.48 ± 18.72	376.83 ± 25.84	291.22 ± 67.32	410.34 ± 6.95	0.5192	0.2146	0.2569
	d 27	258.75 ± 25.55	244.10 ± 19.39	300.27 ± 29.77	320.46 ± 36.00			

Trt, Treatment effect; Trt × Time, Effect of the interaction between treatment and time. Data are presented as means ± standard error.

### 3.7 Intestinal morphology of duodenum and jejunum sections

**Figure 3:**
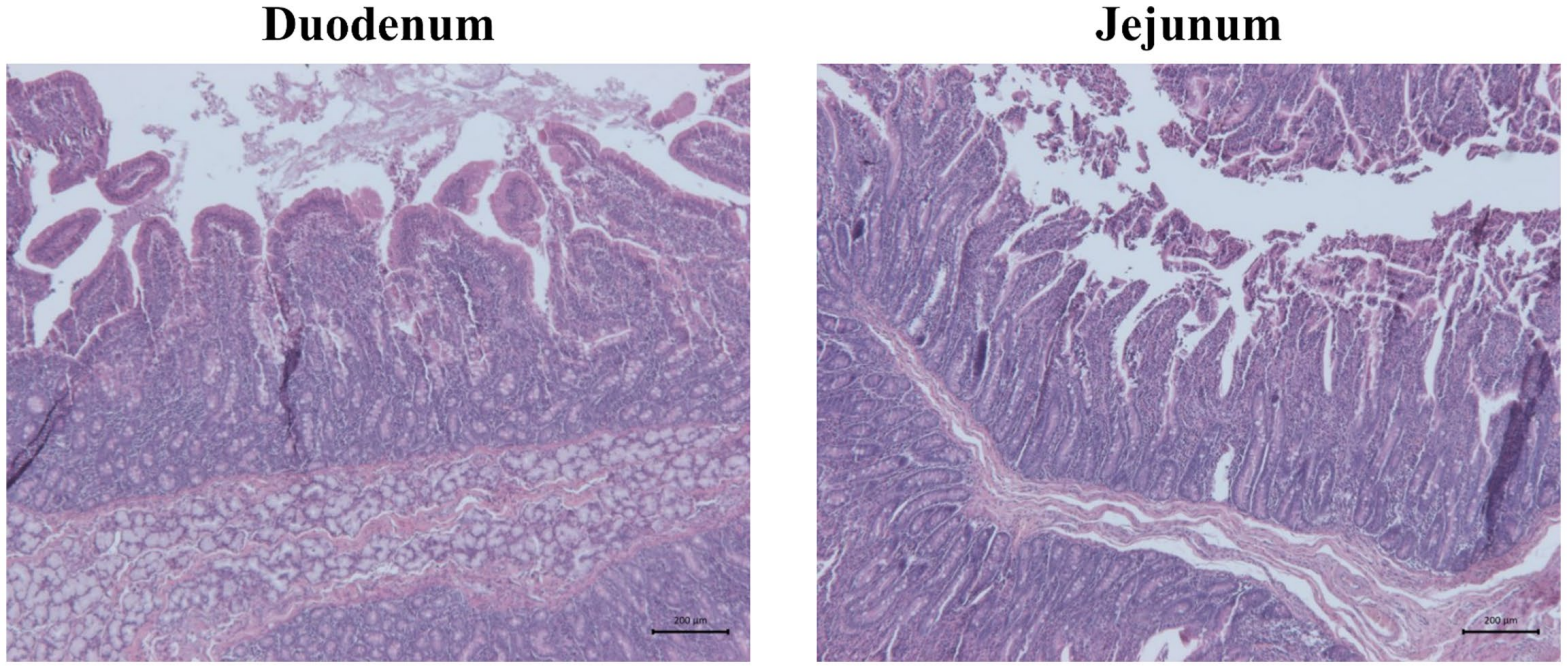
Representative pictures of hematoxylin and eosin staining showing duodenum and jejunum morphology of one piglet of unchallenged control (CTRL−). Scale bar = 200 μm.

The villus height and width showed significant differences in both duodenum and jejunum tracts at 27 days of trial (*p* < 0.001; Table 9; Figure 3). In particular, the villus height was significantly reduced in the duodenum of challenged (CTRL+ and ALGAE+) compared to the unchallenged (CTRL− and ALGAE−) groups (*p* < 0.001; Table 9). Jejunum height showed a significant decrease in CTRL+ compared to other experimental groups. Consequently, the villus height/crypt depth ratio was significantly reduced in CTRL+ compared to other groups (*p* < 0.05).

**Table 9:** Intestinal morphology parameters of duodenum and jejunum sections at 27 days in control (CTRL) and algae mixture (ALGAE) supplemented divided per unchallenged (−) and challenged (+).

Parameter	CTRL−	ALGAE−	CTRL+	ALGAE+	SEM	*p* value
Villus height (μm)						
Duodenum	304.6^a^	285.2^a^	233.2^b^	229.2^b^	8.0	<0.001
Jejunum	328.5^a^	300.4^a^	218.9^b^	294.7^a^	10.3	<0.001
Villus width (μm)						
Duodenum	131.1^a^	135.9^a^	114.5^b^	119.0^b^	3.2	<0.050
Jejunum	118.1^a^	115.6^a^	91.9^b^	119.8^a^	2.8	<0.001
Crypt depth (μm)						
Duodenum	127.0	120.5	109.6	109.7	3.9	ns
Jejunum	97.7	84.8	78.8	92.6	2.7	ns
VH:CD ratio						
Duodenum	2.5	2.7	2.3	2.8	0.2	ns
Jejunum	3.3^a^	3.4^a^	2.7^b^	3.1^a^	0.2	<0.050

VH:CD ratio, Villus height and crypt depth ratio. Data are presented as means ± standard error of the means (SEM). ^a,b^Different lowercase letters indicate statistically significant differences among groups (*p* < 0.05).

## 4 Discussion

In piglets, ETEC *E. coli* cause high occurrence of PWD during the post-weaning period leading to important detrimental effects on animal heath resorting in antibiotic use strictly connected with AMR. Recently, *E. coli* strains are considered important vectors for antimicrobial resistance transmission (39). In our study, ETEC challenge model, using a moderate dosage, was adopted to mimic a biological stressor for evaluating the dietary response of susceptible and non-susceptible pigs under controlled experimental conditions (5). Our study explores the field of functional nutrition in order to propose a multilevel approach to increase animal health and decreasing pathology occurrence thus possibly reducing the antibiotic treatments in swine farming. Algae have demonstrated an interesting profile as nutraceuticals and functional ingredients for in-feed supplementation (21). Particularly, dietary marine algae supplementation have been recognized as source of bioactive compounds to stimulate gut health and performance in post-weaned piglets (40). This study provides valuable insights into the effects of *A. nodosum* and *L. calcareum* supplementation on the growth performance and health of F4+ *E. coli* challenged piglets, with important implications for antibiotic alternatives in swine farming.

Significant differences in growth performance of piglets were observed from day 13 to 27. Particularly, the CTRL+ group exhibited lower BW at day 20 compared to the ALGAE+ group, indicating a potential benefit of algae supplementation in promoting growth after the challenger strain induced stress. The observed lower ADG in CTRL+ from day 13 to 20, followed by a significant weight loss from day 17 to 20, suggested an impairment of homeostasis condition by the challenger strain. Turner et al. (41) reported that supplementation with 1% *A. nodosum* extract did not demonstrate a significant effect on performance over a 28-days trial. Dierick et al. (42) highlighted the inhibitory effect of *A. nodosum* on *E. coli* when tested at 10 g/kg *in vitro*, attributing this protective effect to the content of phlorotannins ranging from 5 to 15% of algae dry weight (43). *A. nodosum* is characterized by the presence of polysaccharides as alginates, fucoidan, laminarin, and polyphenols such as phlorotannins, flavonoids, and pigments (44). Among bioactive compounds of this brown alga, phlorotannins are renewed for their important antimicrobial activity through impairing the cell wall integrity (45). However, Gardiner et al. (46) observed a decrease in ADG in grower-finisher pigs supplemented with 3–9 g/kg of *A. nodosum* extract, suggesting that the presence of phlorotannins may impact feed intake and nitrogen retention, and the high potassium content could lead to an anion-cation imbalance depending on the extract type, while high levels of alginates might increase digesta viscosity. *L. calcareum* can represent a supplementation of a highly soluble source of calcium that constitutes about 35% of its composition (47) that possesses around 2-fold solubility if compared with calcitic limestone (48). This red alga is also a source of magnesium carbonate, iron, zinc, fluorine, manganese, cobalt, iodine, alginates, and vitamin C (49). In addition, *L. calcareum* presents a significant concentration in selenium (≈0.6 ppm) (50) which is involved in glutathione peroxidase could contribute to balance the oxidative stress (51). The observed differences in growth performance among groups could be attributed to the functional components present in *A. nodosum* and *L. calcareum*, which may play a protective role for piglets during stressful weaning conditions. However, marine algae exhibit significant variability in their content of bioactive compounds across batches and seasons. Therefore, it is crucial to characterize the specific bioactive compound profiles of each product to ensure consistency and efficacy in providing beneficial effects under varying environmental and production conditions. In our previous study, the metabolomic profile of *A. nodosum* and *L. calcareum* was determined in terms of polyphenols and tripeptides levels. They were distinguished by their abundant phloroglucinol and oxidized glutathione content, respectively (29). This characterization allowed targeted supplementation strategies for optimizing feed formulation for piglets.

The feed intake did not register significant differences, thus suggesting that the growth disparities were more likely related to nutrient utilization efficiency rather than differences in feed consumption. In addition, the supplementation of 2% of seaweed blend composed by *A. nodosum* and *L. calcareum* supplementation did not impair the feed palatability.

Feed conversion ratio was significantly increased from day 13 to 20 in the CTRL+ group compared to other experimental groups, confirming the detrimental effect of challenge on zootechnical performance during the first week post-challenge. A higher FCR in the absence of algae supplementation may indicate suboptimal nutrient utilization and metabolic stress, possibly due to stress induced by the oral administration of F4+ *E. coli*. In contrast, the lower FCR observed in ALGAE+ and other groups suggests maintained feed efficiency associated with algae supplementation after challenge, which could be linked to enhanced nutrient absorption, gut health, and metabolic balance also under stressful conditions. Dos Santos et al. (48) provided 0.82% of total calcium with *L. calcareum* without registering differences in performance during the pre-starter period. The study disclosed that piglets supplemented with *L. calcareum* registered a significant reduction in FCR during the starter phase. The absence of differences in performance among unchallenged groups suggests that the effects of algae supplementation on growth performance could probably be better exacerbated under challenge conditions. This protective effect was also observed *in vitro* on intestinal cell model stressed with hydrogen peroxide and supplemented with natural extracts (52). Animals challenged and supplemented with seaweed mixture showed similar growth compared to the unchallenged groups. Meanwhile, the CTRL+ group achieved comparable growth at the end of the trial, likely due to the moderate dosage of the challenge. It is important to highlight the potential protective effect of seaweeds that could be observed in field conditions, where the persistence of infection may be higher due to pathogen shedding and reinfections, possibly leading to more significant health implications. Further studies will be necessary to understand the long-term effects of this innovative seaweeds’ dietary supplementation beyond 28 days.

The increase of diarrhea in CTRL group during the first week of pre-challenge was mainly due to the presence of one piglet that showed diarrhea for 5 consecutive days prior to antibiotic treatment and exclusion form experimental trial. This animal contributed for an increase of 1.80% (+4 cases) of diarrhea registrations in CTRL group causing a significant difference for the chi-squared test. Removing this subject form the dataset is it possible only to observe a tendency toward significance for the diarrhea frequency during the first week pre-challenge (9.78 and 5.21% of total observations in CTRL and ALGAE, respectively, *p* < 0.0912). The absence of diarrhea occurrence in both challenged and unchallenged groups from day 13 to 27 is noteworthy. Specifically, the dosage of the experimental challenge was moderated in terms of CFU (53–55) to induce a manageable level of stress without causing clinical diarrhea in piglets during the trial. This approach ensured that the piglets experienced a controlled stressor, while allowing them to develop and mature their immune capabilities. Notably, the challenge study was conducted when the piglets were 48 days old, a period characterized by the ongoing maturation and strengthening of their immune response (56).

The fecal shedding of the challenger strain in challenged piglets, observed as early as 2 days (8.70% of challenged pigs) and 3 days (69.57% of challenged pigs) post-challenge in the ALGAE+ and CTRL+ groups respectively, confirming the success of the oral challenge procedure and suggesting the rapid colonization of the pathogen within the gut during this period. The persistence of F4+ *E. coli* shedding until day 18 underscored the prolonged impact on gut health during this critical period (57). No differences were registered for the fecal count of total, lactic acid and coliform bacteria and the lactic acid/coliform index. While these parameters can be considered as important indicators of gut health, a more comprehensive analysis of the complete gut microbiota will be required to fully elucidate the effects of this innovative algae mixture supplementation. DNA abundance of challenger strain showed a significant increase in feces of ALGAE+ group compared to CTRL+ at day 2, 3, and 4 post-challenge. After 7 days post-challenge (day 20), ALGAE+ registered a significant drop in DNA presence of F4+ *E. coli* in feces compared to CTRL+. This trend could indicate a higher presence of *E. coli* in fecal samples during the first 4 days post-challenge while possibly decreasing the persistence of challenger shedding after 1 week. In particular, the adopted challenge model did not reveal an inhibitory effect in terms of shedding of the challenger strain. We hypothesize that the protection provided by seaweeds was not directly related to the pathogenic component but rather to enhancing the intestinal barrier and immune status of the host.

Generally, piglets that are more resistant to ETEC exposure may show lower shedding levels of F4+ *E. coli* suggesting an absence of significant interaction with the gut and possible competition with the commensal microbiota for the colonization (58). However, after 7 days of challenge the virulence factor prevalence generally decreases possibly negativizing after 10 days from *E. coli* infection (55). As previously reported, the use of natural additives could decrease the fecal shedding of *E. coli* after 2 and 3 weeks of trial (59). Even if no diarrhea was registered during the trial, we can speculate that algae may drop the persistence of challenger strain shedding after 7 days from challenge thus shortening the pathogenic spreading window.

The fecal composition analysis over the 27-day trial period revealed interesting insights into nutrient utilization and protein digestion in response to algae supplementation and experimental challenge. Analyzing the apparent digestibility based on feces is an important parameter to estimate the nutrient efficiency utilization using a minimally invasive method. Currently, there have been few studies describing the impact of seaweeds on diet digestibility. Algae can influence the digestive process in several ways, primarily due to their nutritional composition and bioactive compounds. Generally, seaweeds are known to possibly affect digestion through soluble fibers such as alginate and agar, which can slow down the digestion and absorption of nutrients (60). Seaweeds supplementation appeared to maintain stable the nitrogen utilization. In contrast, the CTRL+ group exhibited a significant increase in fecal crude protein (CP) concentration and a decrease in apparent total tract digestibility (ATTD) of protein at 27 days post-challenge. Even if the biological importance of the current findings can be negligible, these findings could suggest that algae-derived bioactive compounds may contribute to optimizing nutrient utilization and mitigating the negative effects of F4+ *E. coli* challenge on protein digestibility in weaned piglets. In particular, *A. nodosum* is a brown alga rich in alginic acid and fucoidans that could decrease digestibility due to the high content of undigestible fibers. In line with our results, Czech et al. (60) did not register significant differences in crude protein digestibility supplementing 0.6 and 1% of *A. nodosum* from 18 to 64 days of age in weaned piglets. Generally, antioxidant rich algae could protect the digestive tract from oxidative damage, supporting gut health and thereby alleviating digestive discomfort (61). Protein total apparent digestibility indicated the supplementation of 2% of the combination of *A. nodosum* and *L. calcareum* did not negatively affect the nitrogen utilization by animals, one of most important nutrients for piglets’ growth efficiency.

The consistent serum oxidative and antioxidant barrier levels during the experimental period, suggest overall good health without any detrimental effect of challenge at 27 days of trial. On the contrary, Czech et al. (60) registered a significant increase of antioxidant ability of serum, particularly for the activity of catalase, superoxide dismutase and reduced glutathione supplementing 1% of *A. nodosum* in piglets after 46 days of supplementation. The differences detected in oxidative and antioxidant parameters may depend on the content in phytochemicals in the used *A. nodosum* and longer supplementation period compared to our experimental design. Based on previous *in vitro* results obtained by our research group (29), *A. nodosum* and *L. calcareum* combination possess the potential for an enhancing antioxidant ability compared to the singular activity of each alga. However, further studies will be necessary to elucidate the long-term effects of this innovative seaweeds’ combination.

The mRNA transcription in duodenum samples at 27 days did not show significant differences among the four considered groups. In this regard, despite our efforts to minimize variability, the response to the pathogen exhibits significant variation, as evidenced by the obtained data. The multifactorial nature of the disorder suggests that a more consistent response could potentially be observed using specific pathogen-free pigs. Nonetheless, our main goal was to assess the efficacy of seaweeds in mitigating the effects of bacterial challenge under in-field conditions. Another consideration is related to the timing of sample collection. Intestinal samples were obtained at 9 days post-challenge, recognizing that the expression of certain genes, particularly inflammatory cytokines, is predominantly modulated within the initial 24 h following pathogenic invasion (62, 63). Probably further experiments will be necessary to elucidate the effect on mRNA transcription of this innovative combination of marine algae.

The depth of the crypts and the height of the villi are important parameters of intestinal health. In fact, it is known that even a short period of fasting could lead to intestinal alterations and increase intestinal permeability (64). This decrease in barrier strength leads to negative effects on both health and performance (65, 66). In the duodenum, villus height and width were shorter in the challenged compared to unchallenged groups indicating that infection affected intestinal mucosa morphology and the supplementation of macroalgae did not protect from the morphological alterations. However, crypt depth and villus height to crypt depth ratio were not different between all the experimental groups in the duodenum.

In the duodenum, villus height and width were significantly shorter in the challenged compared to unchallenged groups indicating that ETEC *E. coli* affected intestinal mucosa morphology and the supplementation of macroalgae did not protect from the morphological alterations. However, crypt depth and villus height to crypt depth ratio were not different between all the experimental groups in the duodenum. In the jejunum, villus height and width were shorter in the CTRL+ than other groups suggesting that, in this intestinal tract, *E. coli* challenge negatively impacted the gut mucosa. However, in the challenged animals, the supplementation of seaweeds protected the mucosa from morphological alterations. In the jejunum, crypt depth was not different between all experimental groups. However, villus height, width and VH:CD were significantly higher in jejunum of the CTRL- than ALGAE- and ALGAE+ compared to CTRL+. This result indicates the beneficial effect of seaweeds to contrast the negative effect of challenge on gut health. Due to the absence of differences in the jejunum of ALGAE+ with unchallenged groups, it could be speculated that the major protective effects of bioactive compounds of *A. nodosum* and *L. calcareum* combination has been exerted in this intestinal tract. Genova et al. (67) evaluated the intestinal characteristics in piglets fed with *L. calcareum* concluding that this calcium source could be considered an interesting alternative source of calcium, registering improved VH:CD ratio in duodenum. Additionally, the influence of macroalgae on improving the integrity of the intestinal mucosa and increasing villus length contributes to the enhancement of small intestine digestive function and results in better nutrient absorption (60). Algae are also rich in antioxidants, including polyphenols and carotenoids, which help to protect digestive tract cells from oxidative damage, maintain gut health, and reduce inflammation, thereby alleviating digestive discomfort. Given the good health condition of piglets in the ALGAE+ group and the histological parameters observed, we can speculate that algae supported intestinal health as we did not observe inflammation signs or typical mucosal remodeling. In contrast, the CTRL+ group, although not exhibiting clinical signs of diarrhea, showed slightly disturbance of intestinal health correlated with alterations in villi morphology probably due to the challenger strain, likely reflecting ongoing efforts to restore mucosal integrity. These findings highlight the complex interplay between nutritional strategies and gut barrier integrity, emphasizing the need for further investigation into the specific mechanisms underlying these histological changes and their implications for intestinal health and disease resilience. Our data suggest a potential protective effect of this innovative seaweeds’ supplementation on intestinal health and barrier function in challenged piglets, highlighting the importance of further investigations to elucidate the exact mechanisms of action and optimize dietary strategies for promoting gut health in swine farming. Considering the interesting results obtained from the combination of these algae species on piglets health, the combined use of different functional ingredients such as algae should be considered as promising innovative dietary strategy for decreasing the occurrence of multifactorial disorders and the dependency on antibiotics, thereby promoting a more sustainable approach in the livestock industry.

## 5 Conclusion

In conclusion, our study underlines the potential of *A. nodosum* and *L. calcareum* supplementation in supporting animal health and performance in F4+ *E. coli* challenged piglets, with significant implications for reducing antibiotic use in swine farming. Notably, algae supplementation demonstrated the ability to mitigate the adverse effects of *E. coli* challenge, as evidenced by improved growth performance and intestinal health parameters compared to the infected control group. The observed differences in growth performance and nutrient utilization among groups may be attributed to the unique functional components present in these algae species, which could contribute to support piglets during stressful weaning conditions. Additionally, the protective effects on gut barrier integrity further highlight the potential of algae-derived bioactive compounds in promoting gut health and resilience during the post-weaning. Further studies will be necessary to understand the mechanisms and long-term effects of this innovative seaweeds’ supplementation. The obtained findings could contribute to the development of functional dietary strategies to enhance animal health and reduce antibiotic use in livestock.

## Data availability statement

The original contributions presented in the study are included in the article, further inquiries can be directed to the corresponding author.

## Ethics statement

All experimental procedures have been approved by the Animal Welfare Organization of University of Milan and the Italian Ministry of Health (authorization n° 884/2021-PR). The study was conducted in accordance with the local legislation and institutional requirements.

## Author contributions

MD’A: Conceptualization, Data curation, Formal analysis, Investigation, Methodology, Visualization, Writing – original draft, Writing – review & editing. SF: Data curation, Formal analysis, Investigation, Methodology, Writing – review & editing. SR: Data curation, Formal analysis, Investigation, Methodology, Writing – review & editing. IF: Formal analysis, Investigation, Methodology, Writing – review & editing. ES: Formal analysis, Investigation, Methodology, Writing – review & editing. LS: Formal analysis, Investigation, Writing – review & editing. AI: Formal analysis, Investigation, Methodology, Writing – review & editing. FR: Investigation, Methodology, Writing – review & editing. NV: Formal analysis, Investigation, Writing – review & editing. RP: Formal analysis, Investigation, Methodology, Visualization, Writing – review & editing. LR: Conceptualization, Funding acquisition, Investigation, Project administration, Resources, Supervision, Validation, Writing – review & editing.
